# Bilateral Lung Points without Pneumothorax: Exploring “Lung Point” Mimics

**DOI:** 10.24908/pocusj.v10i02.18500

**Published:** 2025-11-17

**Authors:** Michael Dong, Rebecca Davis, F. Mae West, Gillian Naro, Arthur Au, Jonathan Foster, Jillian Cooper

**Affiliations:** 1 Division of Pulmonary and Critical Care Medicine, Thomas Jefferson University Hospital, Philadelphia, PA, USA; 2 Department of Internal Medicine, Thomas Jefferson University Hospital, Philadelphia, PA, USA; 3 Department of Internal Medicine, University of Pennsylvania, Philadelphia, PA, USA; 4 Department of Emergency Medicine, Thomas Jefferson University, Philadelphia, PA, USA

**Keywords:** Lung Ultrasonography, POCUS, Lung point, Mimic, Ultrasound diagnosis of pneumothorax

## Abstract

A “lung point” is the interface between present and absent lung sliding artifacts identified by point of care ultrasound (POCUS). This finding has long been considered highly specific for pneumothorax, with certain studies citing a specificity as high as 100%. More recently, multiple cases that mimic the lung point have been reported. Here, we present a patient case with bilateral lung points in the absence of pneumothorax. We reviewed lung point mimics and explored the conditions that caused this finding without pneumothorax.

## Case Presentation

**Figure 1A, 1B.**
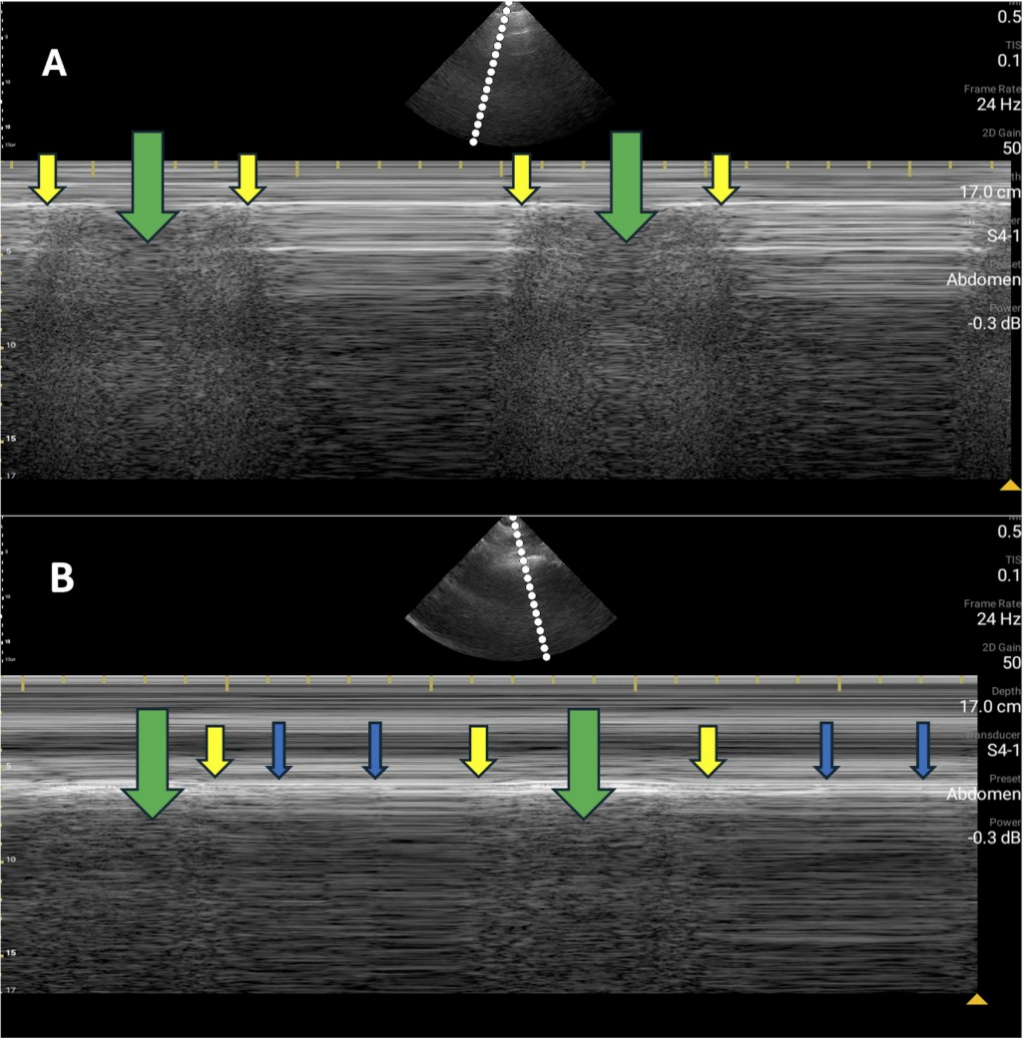
Point of care ultrasound (POCUS) exam in M-mode demonstrating alternating “bar-code sign” and “sandy-shore sign.” Panel A depicts imaging at the left lung apex (at the midclavicular line in the second intercostal space). Panel B depicts imaging at the right axilla (at the right anterior axillary line in the fourth intercostal space). The yellow vertical arrows point to the transition between present and absent lung sliding. The tip of the yellow arrow rests on the pleural line. The green vertical arrows point to normal lung sliding. The blue arrows (Panel B) indicate lung pulse present in the bar-code regions.

**Figure 2.**
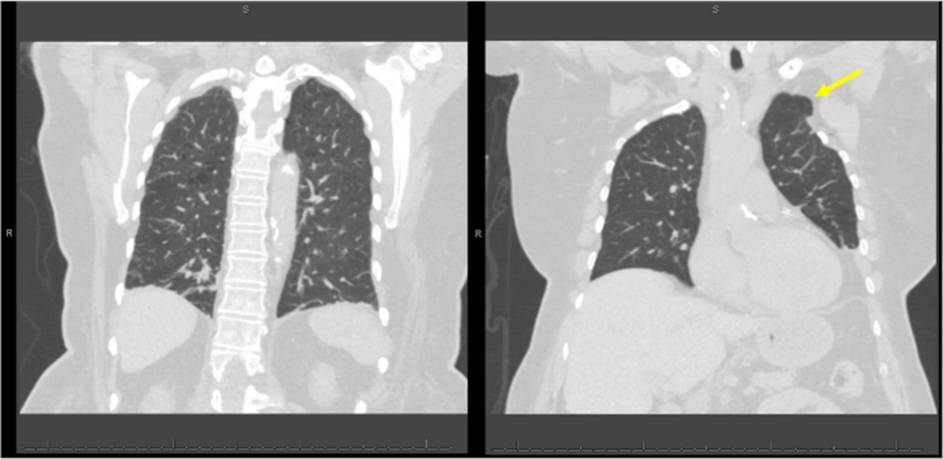
Computed tomography (CT) chest coronal view with emphysema and no evidence of pneumothorax. The yellow arrow is pointing to the left anterior apical lung herniation at the site of prior surgical first rib removal.

An 83-year-old woman presented to the hospital with dyspnea. She had a history of chronic obstructive pulmonary disease (COPD), obstructive sleep apnea, and thoracic outlet obstruction status post left first rib removal 60 years ago. She also reported lower extremity edema, weight gain, and joint pain. Her vital signs included a heart rate of 68 beats per minute, blood pressure of 186/75 mmHg, respiratory rate of 20 breaths per minute, oxygen saturation was 98% on 2 liters per minute of oxygen, and she was afebrile. The patient's physical exam was notable for normal respiratory effort, wheezing and crackles on lung exam, and pretibial edema. A plain chest radiograph reported no focal consolidation, edema, effusion, or pneumothorax. Point of care ultrasound (POCUS) of the lungs demonstrated bilateral lung points (Figures 1A and 1B, Supplementary Materia - S1 and S2) at the left second intercostal space midclavicular line and the right fourth intercostal space anterior axillary line. Scattered B-lines were observed in lung regions outside the focal areas that lacked pleural sliding. A ventilation-perfusion scan was negative for pulmonary embolism. Computed tomography (CT) of the chest was performed to evaluate her dyspnea. It demonstrated emphysematous changes without evidence of pneumothorax, and a small anterior left apical lung hernia at the site of partial left first rib resection (Figure 2). The patient was admitted for a COPD exacerbation.

## Discussion

Lung POCUS has emerged as a reliable tool for the evaluation of pulmonary pathology. POCUS signs such as absent lung sliding and the “lung point” are utilized in the diagnosis of a pneumothorax. The lung point represents the interface between present and absent lung sliding artifacts. Historically, this finding has been considered highly specific or even pathognomonic for pneumothorax, with certain studies citing a specificity as high as 100% [1–3]. However, in conjunction with other recent reports, we challenge the high specificity of lung point for pneumothorax. Here, we present a case of a patient with bilateral lung points in the absence of pneumothorax. We further explore the pathophysiologic mechanisms underlying lung point findings without pneumothorax and how these mimics arise.

Lung point was first described by Lichtenstein in 2000 as “the fleeting appearance of a lung pattern (lung sliding or pathologic comet-tail artifacts) replacing a pneumothorax pattern (absent lung sliding plus exclusive horizontal lines).” It was reported to have a sensitivity of 66% and specificity of 100% when the ultrasonography findings were compared to CT imaging that verified the presence or absence of pneumothorax [2].

Recently, several reports have identified lung point mimics that exhibit similar POCUS findings to the lung point seen in pneumothorax. These mimics arise at the intersection between normal lung tissue and pathological or anatomical structures that abolish or obscure lung sliding [4]. For example, a physiologic sign in a healthy lung that mimics a lung point can be visualized when the lingula slides over the heart during respiration [5]. This finding occurs at the mediastinal pleura, where contact exists between the visceral pleura and the mediastinal soft tissue. Additionally, the term “pseudo-lung point” has been used to describe the interface between lung sliding and the absence of lung sliding over the site of a pulmonary contusion between contused and aerated lung [6]. In 2015, the term “bleb point” was introduced to describe a lung point mimic at the border between a bulla and the aerated lung tissue in cases of bullous lung disease [7]. It has been postulated that the bleb point is caused by advanced bullous lung disease that leads to thinning of the visceral pleura to the point where lung sliding between the pleural layers is obscured on POCUS examination [7,8]. Severe obstructive lung diseases can mimic an absence of lung sliding due to a combination of air trapping, hyperinflation, and decreased movement of the pleura that make it difficult to detect lung sliding, especially at the lung apices. This mechanism is similar to that seen in the bleb point phenomenon above [9]. There are conditions in which lung sliding is absent due to an inflammatory effect that fixes the opposing pleural layers. The 2008 BLUE Protocol includes pneumonia on the differential when lung sliding is abolished [10]. Prior thoracic surgery and pleurodesis can also cause an absence of lung sliding, as fibrosis between the parietal and visceral pleura are induced [11].

In the case described above, bilateral lung points were observed at the lung apices in a patient with COPD, emphysema, and a history of intrathoracic surgery, with no evidence of pneumothoraces on CT imaging. Lung herniation at the site of the left first rib resection may have disrupted lung sliding, potentially mimicking a lung point on the left side. Although the patient had mild emphysema, there was no extensive bullous disease to account for a bleb point mimic. POCUS evaluation with M-mode revealed the characteristic “bar-code sign,” which, in the context of pneumothorax, results from a lack of contact or movement between the visceral and parietal pleura. This alternates with the normal “sandy-shore sign” and is the classic description of the lung point. A notable finding within the M-mode imaging is the presence of an intact lung pulse in the bar-code sign regions (Figure 1B, blue arrows).

The lung pulse is a dynamic POCUS finding caused by the transmission of cardiac pulsations to the pleural layers and pulmonary parenchyma [12]. It is most prominent when the lung is adjacent to the heart or in cases of poor aeration or atelectasis, as less aerated pulmonary tissue more effectively transmits the cardiac impulse. The presence of a lung pulse confirms that the visceral and parietal pleura are closely apposed to the chest wall, allowing the transmission of cardiac impulses from the heart, through the chest wall, to the lung. Since a true pneumothorax requires pleural separation by air, a lung pulse should not coexist with a pneumothorax [3]. However, a lung pulse may appear adjacent to the lung point if impulse transmission occurs in a region where the pleural layers remain adherent at the margin of the pneumothorax [13]. In our case, lung pulse is present within the bar-code sign regions at the right axilla, which should not be possible in a true pneumothorax. This paradoxical observation further supports the interpretation of a lung point mimic. It also supports the use of the lung pulse as a distinguishing feature to differentiate a lung point caused by pneumothorax from one created by alternative pathologies, such as pleural adhesions or fibrosis. Interestingly, we did not observe lung pulse in the M-Mode tracing at the left apex, which may be due to post-surgical changes.

In addition to using B-mode and M-mode imaging to identify lung sliding, the presence of some POCUS findings can rule out pneumothorax. A common feature among these findings is that they occur only when the pleural layers remain apposed. B-lines are vertical, hyperechoic artifacts generated at the interface between air-filled alveoli and fluid-filled interstitial spaces. Physiologic B-lines may also occur due to lung fissures, micro-atelectasis, and other non-pathologic states. Regardless of etiology, their presence confirms pleural apposition and indicates that the ultrasound signal is reaching the lung visceral pleura, effectively ruling out pneumothorax at that location [1]. In our case, a B-line is visible in B-mode imaging adjacent to the lung point, which can occur if an alveolar interstitial syndrome is present near the pneumothorax margin. However, since B-lines should not appear within a pneumothorax, their presence should prompt reconsideration of the diagnosis and further imaging in the area of interest. Lung pulse occurs due to vibrations at the pleural lines caused by cardiac activity and necessitates the pleural layers be apposed; it should not be present within a pneumothorax, as discussed above [12]. Although less commonly reported, the addition of color Doppler at the pleural line can detect subtle pleural motion and has been described as the “power slide” technique [14,15]. This application has limitations due to the high sensitivity of color flow Doppler, as even minor movements from the patient or the probe—such as chest wall expansion during respiration—can produce false positives. Once the absence of lung sliding is established, differentiating a true lung point from its mimics may depend on the presence or absence of these additional imaging features. Of utmost importance is the indication and clinical context in which the lung POCUS is being performed. The unexpected finding of a lung point should prompt consideration of its mimics.

In the patient described above, while prior thoracic surgery and lung herniation could potentially account for the lung point finding on the left side, we theorize that pleural adhesions may be responsible for the bilateral findings that were observed in our patient. We suspect that pleural adhesions may represent an underappreciated confounding factor in lung POCUS interpretation. Adhesions are challenging to detect on cross sectional imaging, particularly those measuring less than 1 mm in thickness, which may necessitate high-resolution CT imaging for visualization [16]. Fixation of the visceral and parietal pleura at adhesion sites can result in absent lung sliding between the pleural layers. Ultrasound imaging at the interface between the surrounding normal lung tissue and the pleural adhesion may produce a transition point characterized by absent lung sliding, mimicking a lung point [17,18]. However, in contrast to a true lung point caused by pneumothorax, this lung point mimic caused by adhesions could be associated with lung pulse and B-lines in the area of interest. Conditions such as lung cancer, pleural infections, prior thoracic surgery, COPD, connective tissue diseases that cause pleurisy, radiation therapy, and asbestos exposure are likely to predispose a patient to pleural adhesion formation, although the exact incidence and prevalence remains undefined [16,19–21].

In conclusion, pleural adhesion points, along with the other lung point mimics described above, should be considered when a lung point is identified on POCUS. Patients with a history of COPD, emphysema, prior thoracic surgery, fibrosing or inflammatory pleural conditions, or lung cancer may exhibit pleural adhesion points, bleb points, and other mimics that can lead to diagnostic confusion. The specificity of the lung point for pneumothorax is likely far lower than previously reported, with an increased risk of false positives, particularly in the patient populations mentioned above who often present with dyspnea. Further research is needed to ascertain the true specificity of lung point for pneumothorax. The diagnosis of pneumothorax using POCUS requires a comprehensive assessment of multiple findings beyond lung sliding, including B-lines, lung pulse, and the power slide technique—all of which help distinguish true pneumothorax from potential mimics. Accurate interpretation depends not only on recognizing these imaging features and mimics, but also on integrating them within the broader clinical context to avoid misdiagnosis and guide appropriate management.
